# Caffeic acid phenethyl ester suppresses androgen receptor signaling and stability via inhibition of phosphorylation on Ser81 and Ser213

**DOI:** 10.1186/s12964-019-0404-9

**Published:** 2019-08-20

**Authors:** Ying-Yu Kuo, Chieh Huo, Ching-Yu Lin, Hui-Ping Lin, Jai-Shin Liu, Wen-Ching Wang, Chuang-Rung Chang, Chih-Pin Chuu

**Affiliations:** 1 Institute of Cellular and System Medicine, National Health Research Institutes, Room R2-2021, 35, Keyan Road, Zhunan Town, Miaoli County 35053 Taiwan; 2 Institute of Biotechnology, National Tsing Hua University, Room 506, LS Bldg. II, Hsinchu City, 30013 Taiwan; 3 Department of Biotechnology and Pharmaceutical Technology, Yuanpei University of Medical Technology, Hsinchu City, 30015 Taiwan; 4 Institute of Molecular & Cellular Biology, National Tsing Hua University, Hsinchu City, 30013 Taiwan; 5 PhD Program for Aging and Graduate Institute of Basic Medical Science, China Medical University, Taichung City, 40402 Taiwan; 6 Biotechnology Center, National Chung Hsing University, Taichung City, 40227 Taiwan

**Keywords:** Caffeic acid phenethyl ester, AR, CDK1, AKT, Prostate cancer

## Abstract

**Background:**

Androgen receptor (AR) plays important role in the development, progression, and metastasis of prostate cancer (PCa). Caffeic acid phenethyl ester (CAPE) is the main component of honey bee propolis. We determined if CAPE affects the signaling and stability of AR in PCa cells.

**Methods:**

Effects of CAPE on AR transcriptional activity and localization were determined by reporter gene assay and immunofluorescent microscopy. Western blotting, fluorescent polarization, computer simulation, and animal experiment were performed to investigate the molecular mechanism how CAPE reduces the stability of AR.

**Results:**

CAPE treatment dose-dependently suppressed the transcriptional activity of AR as well as the protein levels of AR and its target gene PSA. Cyclohexamide treatment revealed that androgen stabilized AR protein, but AR stability was diminished by CAPE. Fluorescence microscopy demonstrated that androgen promoted the nucleus translocation of AR in PCa cells, while treatment with CAPE reduced protein level of AR in both nucleus and cytoplasm. CAPE treatment suppressed the phosphorylation of Ser81 and Ser213 on AR, which regulates the stability of AR. CDK1 and AKT are the kinases phosphorylating Ser81 and Ser213 on AR, respectively. CAPE treatment significantly reduced the protein level and activity of CDK1 and AKT in PCa cells. Overexpression of CDK1 or AKT rescued the AR protein level under CAPE treatment.

**Conclusions:**

Our results suggested that CAPE treatment reduced AR stability and AR transcriptional activity in PCa cells, implying the possibility of using CAPE as a treatment for advanced PCa.

**Graphical abstract:**

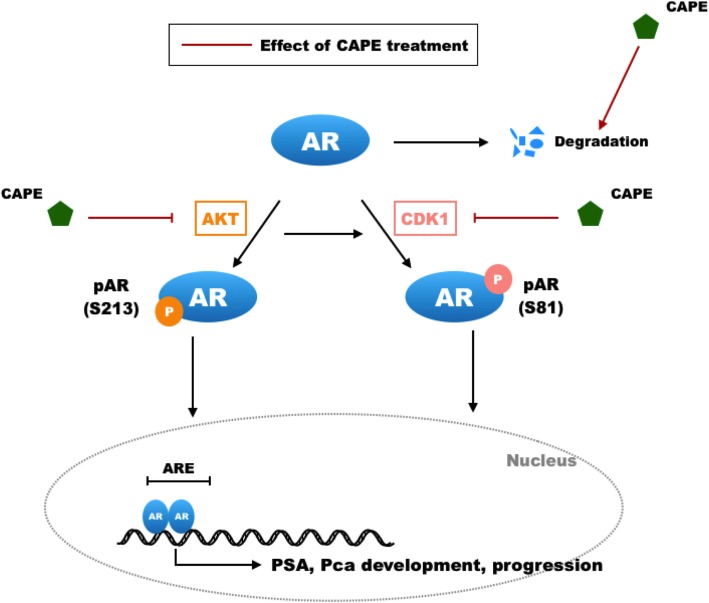

**Electronic supplementary material:**

The online version of this article (10.1186/s12964-019-0404-9) contains supplementary material, which is available to authorized users.

## Background

Androgen receptor (AR) is an androgen-activated transcription factor belongs to the nuclear receptor superfamily [1]. Binding of androgen to AR induces dissociation of AR from heat-shock proteins (HSPs) and stimulates AR phosphorylation [2]. AR dimerizes, translocates into the nucleus, and binds to androgen-response elements (ARE) in the promoter regions of target genes [2]. Co-activators and co-repressors bind the AR complex, facilitating or preventing the transcription of AR target genes, which regulate the growth, survival, and the production of prostate specific antigen (PSA) in prostate cells [3, 4].

AR regulates male sexual maturation, maintenance of normal prostate function, prostate carcinogenesis, and prostate cancer (PCa) progression [4, 5]. AR plays important role in the development, progression, and metastasis of PCa [2, 6, 7] and AR modulates the expression of proteins regulating cell cycle, survival, and growth [8–10]. Androgen ablation therapy is the primary treatment for metastatic PCa. However, a majority of PCa patients receiving the androgen ablation therapy will ultimately develop recurrent castration-resistant prostate cancer (CRPC) within 1–3 years after treatment with a median overall survival time of 1–2 years after relapse. Increase in AR mRNA and protein were observed in nearly one-third of patients developing CRPC [11–13]. Increase in AR mRNA and protein were found to be necessary and sufficient to convert PCa growth from a hormone-sensitive to a hormone-refractory stage [4, 14, 15]. Abiraterone acetate and enzalutamide, the two androgen receptor (AR) pathway inhibitor drugs used for advanced PCa, have been hindered by the emergence of drug resistance [16]. As a result, compounds induce degradation of AR protein may be a novel therapeutic agent for advanced PCa.

Caffeic acid phenethyl ester (CAPE), a strong antioxidant, is the major bioactive component in honeybee hive propolis [17, 18]. CAPE is a specific NF-κB inhibitor [18]. Our previous studies indicate that CAPE treatment suppresses proliferation, migration, and invasion of PCa cells [19–21]. As AR regulates the proliferation and metastasis of PCa cells, we investigate if CAPE treatment interferes the activity and expression of AR in the present study.

## Materials and methods

### Cell culture, chemicals and plasmids

LNCaP 104-S and LNCaP 104-R1 cells were generated from ATCC FGC clone (ATCC CRL-1740) as described in previous publication [22]. LNCaP C4–2 cell line is gift from Dr. Hsing-Jien Kung (NHRI, Taiwan). LNCP FGC, LNCaP 104-S, and LNCaP C4–2 cells were maintained in DMEM medium supplemented with 10% FBS (Gibco/Thermo Fisher Scientific, Waltham, MA, U.S.A) and 1 nM DHT (Sigma Aldrich, St. Louis, MO, U.S.A). LNCaP 104-R1 was maintained in DMEM medium supplemented with 10% CS-FBS (charcoal stripped fetal bovine serum) [22, 23]. Condition medium of LNCaP 104-S cells was replaced with 10% CS-FBS medium for 72 h before experiments. HEK293-AR cells were generated from Human embryonic kidney 293 (HEK293) cells transfected with SG5 plasmid containing wild type AR and were being selected with hygromycin. AKT overexpression in LNCaP 104-S and LNCaP 104-R1 cell lines has previously been described [20]. For re-expression of AR in AR-negative PC-3 cells, PC-3 cells were transfected with LNCX-2 plasmid containing wild-type human AR and selected with neomycin G418 as previously described [24]. Antibiotic-resistant colonies were expanded. PC-3 cells overexpressing AR were denoted as PC-3^AR^. PC-3^AR^ cells were maintained in DMEM (Gibco/Invitrogen) supplemented with 10% charcoal-stripped fetal bovine serum (CS-FBS) (FBS was purchased from Atlas Biologicals, Fort Collins, CO, U.S.A.), penicillin (100 U/ml), and streptomycin (100 μg/ml). Caffeic acid phenethyl ester (CAPE) was purchased from Sigma Aldrich. Cyclohexamide was purchased from Calbiochem/Merck Millipore (Burlington, MA, U.S.A).

### Dual luciferase assay

Cells were seeded in 12-well plates at the density of 2.5 × 10^5^ cells per well. After 24 h, pRL-TK (rellina luciferase vector for normalization, 0.75 ng/well) and p3xARE-∆56-c-Fos-GL3 (reporter gene vector) were co-transfected by using PolyJet in vitro DNA transfection reagent (SigmaGen Laboratories) for 5 h, and substituted medium containing DHT or/and CAPE for 48 h. Cell lysates were lysed in 100 μl 1X passive lysis buffer (Promega). Dual-luciferase reporter assay kit (Promega) was used to measure transcriptional activity by Turner Biosystems 20/20n Luminometer.

### Immunoblot analysis

Cell lysate was lysed and Western blot was performed as previously described [20]. Antibodies against AR was purchased from Abcam (Cambridge, MA, U.S.A). The phospho-AR Ser81 and Ser308 antibody were purchased from Millipore and Santa Cruz (Dallas, TX, U.S.A). PSA antibody was purchased from DAKO/Agilent (Santa Clara, CA, U.S.A). Phospho-AR S213 and Lamin A/C antibodies were purchased from GeneTex (Irvine, CA, U.S.A). Antibody against CDK1, CDK5, CDK9, Cyclin B1, AKT, phospho-AKT Ser473, phospho-AKT Thr308 were purchased from Cell Signaling (Danvers, MA, U.S.A). β-actin and GAPDH antibody were purchased from Novus (Littleton, CO, U.S.A). Antibody against IgG was purchased from Santa Cruz (Dallas, TX, U.S.A). The intensity of indicated Western blot bands were quantified by ImageJ software.

### Real-time polymerase chain reactions

Cell lysate were prepared for RNA extraction using RNeasy mini kit (Qiagen, Venlo, Netherlands). Two micrograms of total RNA of each samples was used as templates for synthesis of complementary DNA (cDNA) by RevertAid H Minus First Strand cDNA Synthesis Kit (Thermo Scientific). qPCR analysis was performed by Maxima SYBR Green/ROX qPCR Master Mix (2X) (Fermentas/Thermo Fisher Scientific). The mRNA expression was analyzed by ABI PRISM 7500 (Applied Biosystems/Life Technologies, Carlsbad, CA, U.S.A). The following sequences were used as qPCR primers: AR-Fw: CTGAAACTACAGGAGGAAGG, AR-Rv: TGCAGAGGAGTAGTGCAGAG; PSA-Fw: CATCAGGAACAAAAGCGTGAT, PSA-Rv: AGCTGTGGCTGACCTGAAATA; CDK1-Fw: CTGGGGTCAGCTC GTTACTC, and CDK1-Rv: TCCACTTCTGGCCACACTTC. GAPDH-Fw: ACAGT CAGCCGCATCTTCTT and GAPDH-Rv: ACGACCAAATCCGTTGACTC.

### Immunofluorescence

Cells were seeded in 35 mm imaging dish (ibidi), and fixed with 4% formaldehyde on ice for 15 min and permeabilized in 0.3% Triton X-100 (in PBS) for 10 min. Blocking for an hour and cells stained with indicated antibody for 16 h at 4 °C. Alexa Fluor 488 dye (Thermo Fisher Scientific) was used as secondary antibody for green-fluorescent dye. The cell nuclei were stained by DAPI. The image of fluorescence was taken by Leica TCS SP5 AOBS Confocal Spectral Microscopy using a 63x oil-immersion objective len and a 10x eyepiece. A scale bar showing 25 μm was arranged at the lower right part of each image.

### Nuclear and cytosolic extraction

Cells were lysed in lysis buffer (50 mM Tris, 5 mM MgCl_2_, 0.4% NP-40, pH 7.5) and centrifuged for 2 min at 3000 rpm at 4 °C. Removing the supernatant and re-suspending the pellet by lysis buffer. Centrifuging for 2 min at 3000 rpm and collecting the supernatant in new eppendorf (cytosol fraction). The pellet was re-suspended for 15 min on ice in nuclear extraction buffer (20 mM HEPES pH 7.9, 25% glycerol, 420 mM NaCl, 1.5 mM MgCl_2_, 0.2 mM EDTA and 0.5 mM DTT) and nuclear fraction was collected by centrifugation for 10 min at 13000 rpm at 4 °C.

### Data analysis and sample size

Data are presented as the mean +/− SD of at least three experiments or are representative of experiments repeated at least three times. Student’s t test (two-tailed, unpaired) was used to evaluate the statistical significance of results from proliferation assay experiments.

## Results

### CAPE inhibits AR transcriptional activity

**Fig. 1:**
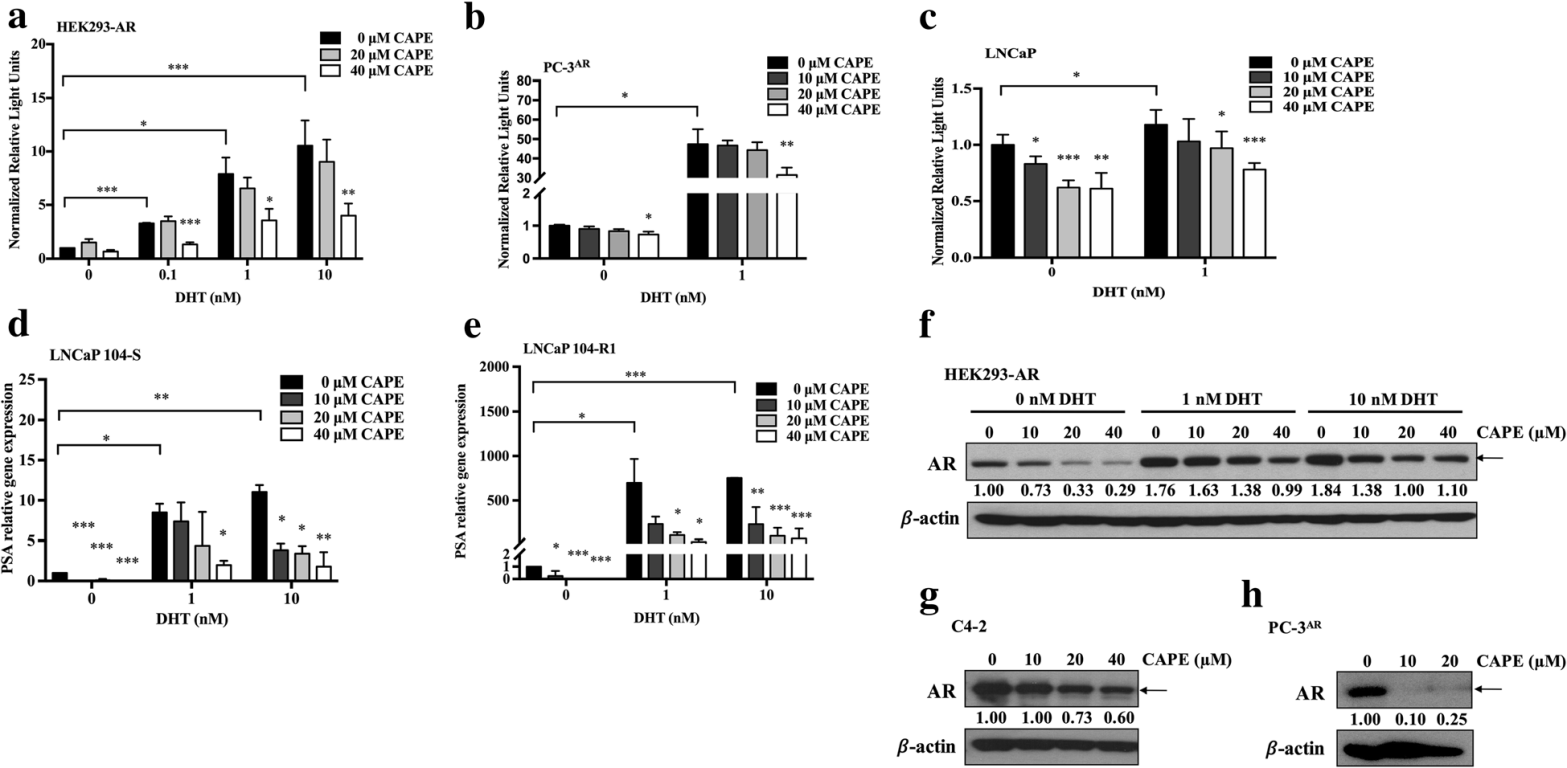
CAPE treatment suppressed transcriptional activity of androgen receptor (AR). The pRL-TK-Renilla luciferase plasmid and p3xARE-∆56-c-Fos-GL3 reporter gene plasmid were co-transfected into HEK293 cells constitutively expressing AR (HEK293-AR) for 5 h, and cells were then treated with increasing concentration of DHT (0, 0.1, 1, 10 nM) and CAPE (0, 20, 40 μM) for 48 h. AR transcriptional activity in HEK293-AR cells (**a**), PC-3^AR^ cells (**b**), or LNCaP FGC cells (**c**) was then determined by luciferase-reporter gene assay. Gene expression level of PSA in LNCaP 104-S cells (**d**) and LNCaP 104-R1 cells (**e**) treated with increasing concentration of DHT (0, 1, 10 nM) and CAPE (0, 10, 20, 40 μM) for 48 h was determined by qRT-PCR. GAPDH was used as loading control. Asterisks *, **, and *** represented statistical significance *p* < 0.05, *p* < 0.01, and *p* < 0.001, respectively, between the treatment group and the control group. AR protein level in HEK293-AR cells (**f**), LNCaP C4–2 cells (**g**), and PC-3^AR^ cells (**h**) treated with indicated concentration of DHT or CAPE for 48 h was determined by Western blotting assay. The numbers under the blot represented the protein level of AR normalized to the loading control β-actin

To determine if CAPE treatment interferes AR transcriptional activity, we expressed AR in human embryonic kidney (HEK) 293 cells, which is AR-negative. Luciferase reporter gene assay indicated that dihydrotestosterone (DHT) stimulated AR transcriptional activity dose-dependently while CAPE treatment suppressed AR transcriptional activity in HEK293-AR cells (Fig. 1a). Treatment with 40 μM CAPE decreased 60% of AR’s transcriptional activity in the presence of 10 nM DHT (Fig. 1a). CAPE treatment also suppressed AR transcriptional activity in PC-3^AR^ cells (AR-negative PC-3 cells being overexpressed of wild type AR) (Fig. 1b) and LNCaP FGC cells (Fig. 1c). LNCaP cells have a mutation T877A on their AR. Our observation suggested that the T877A mutation does not affect the suppressive effect of CAPE on AR activity.

PSA is a target gene of AR. As CAPE treatment reduces AR transcriptional activity, we predict that CAPE treatment can suppress PSA expression in human PCa cells. We determined the mRNA level of PSA in androgen-dependent AR-positive LNCaP 104-S cells and androgen-independent AR-rich LNCaP 104-R1 cells in the presence or absence of androgen. Our qRT-PCR data revealed that while androgen stimulated the expression of PSA mRNA, CAPE treatment suppressed the androgen-induction of PSA mRNA in both LNCaP 104-S (Fig. 1d) and 104-R1 (Fig. 1e) cell lines.

### CAPE suppresses AR protein level but not mRNA

**Fig. 2:**
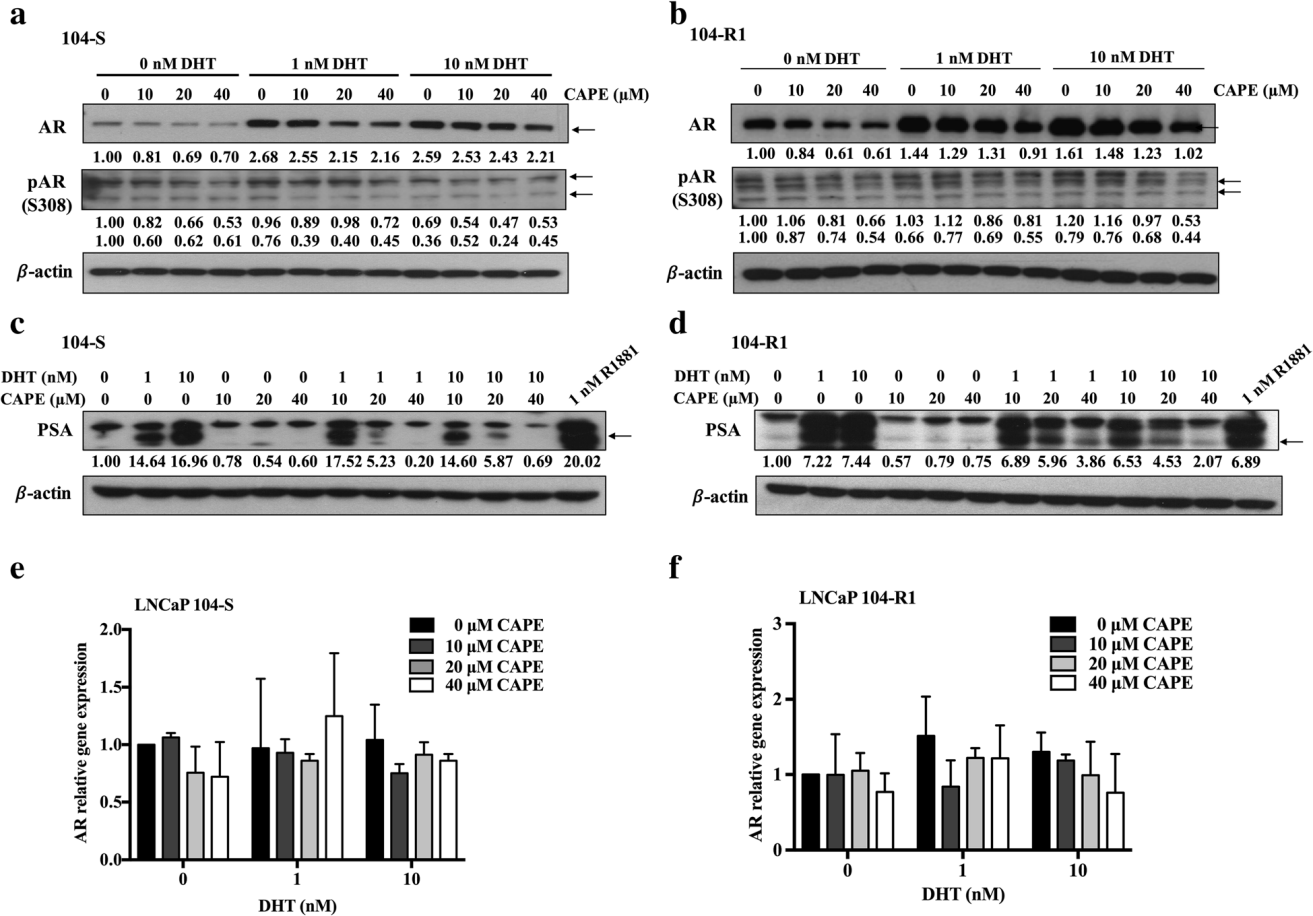
CAPE treatment suppressed protein level of AR and PSA but not mRNA of AR. Protein expression level of AR, phospho-AR Ser308 in LNCaP 104-S cells (**a**) and 104-R1 (**b**) cells as well as PSA in in LNCaP 104-S cells (**c**) and LNCaP 104-R1 cells (**d**) treated with DHT and CAPE for 48 h was determined by Western blotting. The β-actin was used as loading control. The mRNA expression level of AR in LNCaP 104-S cells (**e**) and LNCaP 104-R1 cells (**f**) treated with indicated concentration of DHT and CAPE for 48 h was analyzed by qRT-PCR. GAPDH was used as loading control

We next examined if CAPE inhibits AR signaling via reduction of either mRNA and protein level of AR. We observed that AR protein expression level in HEK293-AR cell line was induced by androgen, but it was dose-dependently inhibited by CAPE (Fig. 1f). CAPE also suppressed the protein abundance of AR in LNCaP C4–2 cells (Fig. 1g) and PC-3^AR^ cells (Fig. 1h). Androgen treatment induced protein expression of both AR and PSA, while CAPE treatment dose-dependently suppressed the protein level of AR (Fig. 2a, b) and PSA (Fig. 2c, d) in LNCaP 104-S and LNCaP 104-R1 cells. Surprisingly, CAPE treatment did not affect AR mRNA level in LNCaP 104-S (Fig. 2e) and LNCaP 104-R1 cells (Fig. 2f), suggesting the possibility that CAPE treatment reduces AR protein stability. Phosphorylation of the AR on Ser308 by CDK1 during mitosis regulates the localization and transcriptional activity of AR [25]. We therefore determined if CAPE treatment affects the phosphorylation of AR. Indeed, CAPE treatment dose-dependently reduced the phosphorylation of Ser308 on AR in both LNCaP 104-S and LNCaP 104-R1 cells (Fig. 2a, b).

### CAPE reduces the abundance of AR protein in cytoplasm and nucleus

**Fig. 3:**
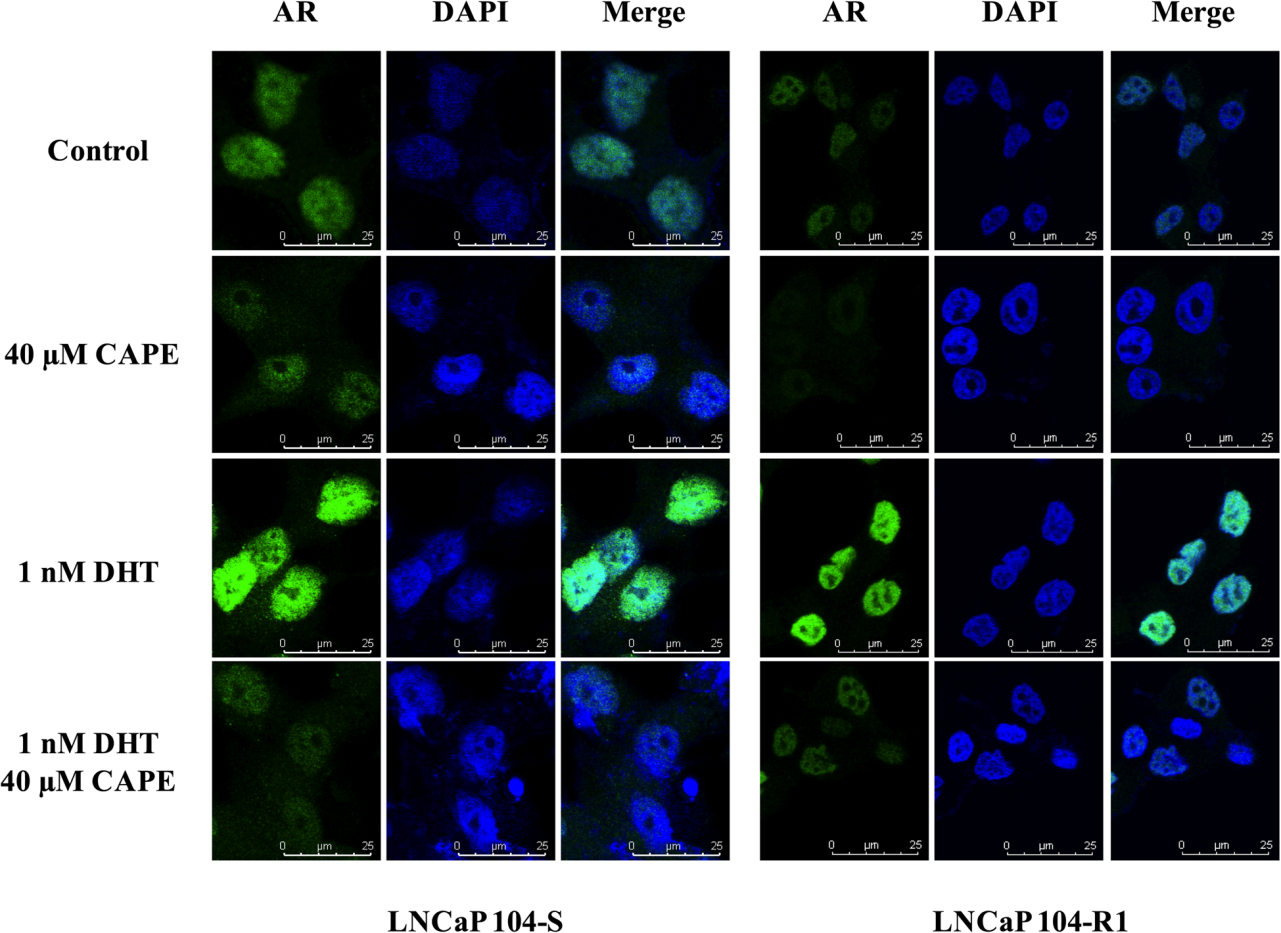
The distribution of AR in LNCaP 104-S and 104-R1 cells treated with or without androgen and CAPE. LNCaP 104-S and LNCaP 104-R1 cells were treated with or without 1 nM DHT and 40 μM CAPE for 48 h. Distribution of AR and nucleus was monitored by immunofluorescence staining using Leica TCS SP5 AOBS Confocal Spectral Microscopy with green and blue fluorescence, respectively. A 63x oil-immersion objective len and a 10x eyepiece were used. A scale bar showing 25 μm was arranged at the lower right part of each image

**Fig. 4:**
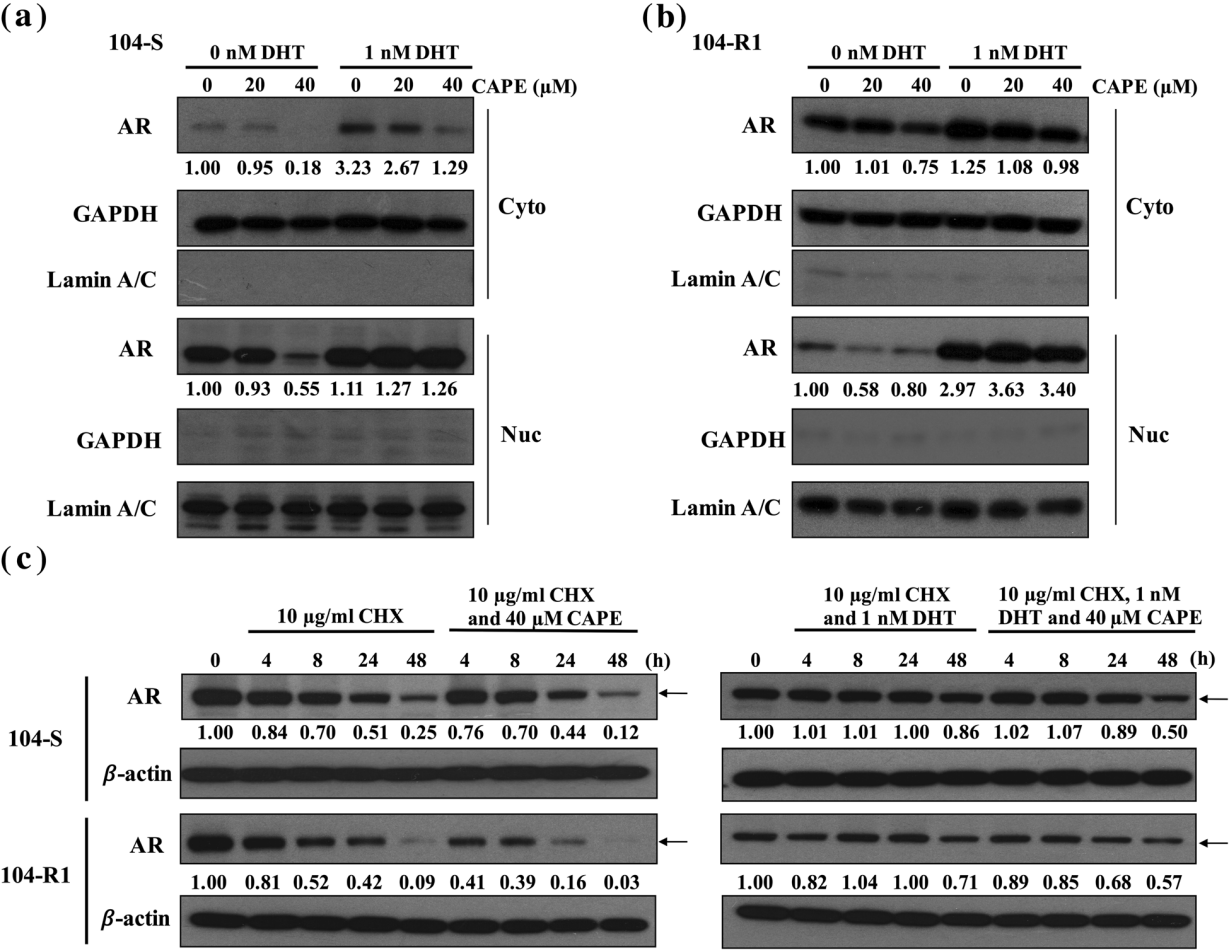
CAPE treatment suppressed AR protein level by accelerating the degradation of AR. Protein abundance of AR in nucleus and cytoplasm of LNCaP 104-S (**a**) and LNCaP 104-R1 (**b**) cells being treated with or without DHT and increasing concentration of CAPE for 48 h was determined by Western-blotting. GAPDH and lamin A/C were used as loading control for cytoplasmic and nuclear extract, respectively. LNCaP 104-S and LNCaP 104-R1 (**c**) cells were treated with 10 μg/ml cycloheximade (CHX) plus 40 μM CAPE or/and 1 nM DHT for 4, 8, 24, and 48 h. AR protein level was determined by Western blotting

We further examined the AR distribution in LNCaP 104-S and LNCaP 104-R1 cells under the treatment of DHT or CAPE. Treatment with DHT increased the AR protein expression and promoted the nuclear translocation of AR (Fig. 3). CAPE treatment reduced protein abundance and nuclear accumulation of AR in LNCaP 104-S and LNCaP 104-R1 cells (Fig. 3). Cytoplasmic and nuclear extraction analysis demonstrated that CAPE treatment reduced abundance of AR in cytoplasm and nucleus of LNCaP 104-S (Fig. 4a) and LNCaP 104-R1 cells (Fig. 4b), while 1 nM DHT antagonized the suppressive effect of CAPE (Fig. 4a, b).

### CAPE accelerates AR protein degradation by inhibiting CDK1 activity, AKT activity and the phosphorylation of AR

**Fig. 5:**
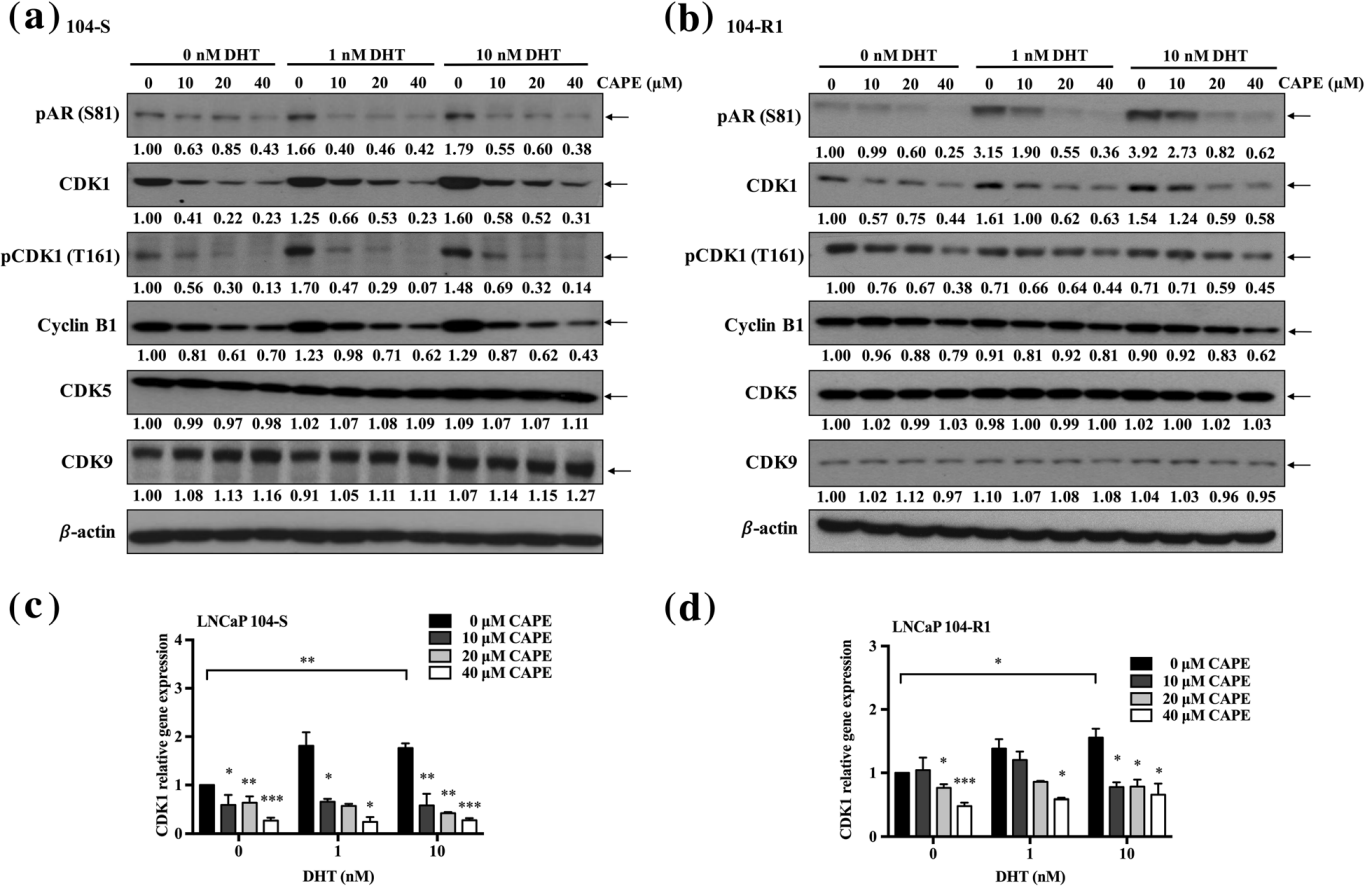
CAPE treatment suppressed phosphorylation of AR on Ser81 and expression level of CDK1. Protein level of phospho-AR Ser81, CDK1, phospho-CDK1 T161, Cyclin B1, CDK5, and CDK9 in LNCaP 104-S (**a**) and 104-R1 (**b**) cells treated with DHT (0, 1, 10 nM) and CAPE (0, 10, 20, 40 μM) for 48 h was determined by Western blotting. The mRNA level of CDK1 in LNCaP 104-S (**c**) and LNCaP 104-R1 cells (**d**) treated with indicated concentration of DHT and CAPE for 48 h was analyzed by qRT-PCR. GAPDH was used as loading control. Asterisks *, **, and *** represented statistical significance *p* < 0.05, *p* < 0.01, and *p* < 0.001, respectively, between the treatment group and the control group

To determine if CAPE affects AR stability, we treated LNCaP 104-S and LNCaP 104-R1 cells with or without CAPE and dihydrotestosterone (DHT), in the presence of cycloheximide (CHX) for 48 h. DHT stabilized AR protein, while CAPE promoted the degradation of AR protein (Fig. 4c). In the presence of DHT, CAPE partially blocked the effect of DHT on stabilizing AR proteins. As AR signaling and stability is regulated by phosphorylation, we determined if CAPE treatment reduces the phosphorylation of AR. Treatment with DHT increased the phosphorylation of AR on Serine 81 in both LNCaP 104-S (Fig. 5a) and LNCaP 104-R1 cells (Fig. 5b). On the other hand, CAPE treatment dose-dependently reduced the phosphorylation of AR on Ser81 (Fig. 5a, b). Cyclin-dependent kinase 1 (CDK1), CDK5, and CDK9 have previously been reported to regulate the phosphorylation of AR on Ser81. We therefore examined if CAPE treatment affects the protein level of CDK1, CDK5, and CDK9. CAPE treatment dose-dependently reduced CDK1 protein, but not CDK5 and CDK9 protein in both LNCaP 104-S (Fig. 5a) and LNCaP 104-R1 (Fig. 5b) cells. Phosphorylation of CDK1 on Thr161, which stimulates the kinase activity of CDK1 [26], was also suppressed by CAPE treatment (Fig. 5a, b). Activation of cyclin B1-Cdk1 complex contributes to the separation of centrosomes in late G2, which is important for mitotic cell division and chromosome separation. CAPE treatment also suppressed cyclin B1 (Fig. 5a, b). Additionally, CAPE decreased the mRNA level of CDK1 in both LNCaP 104-S (Fig. 5c) and LNCaP 104-R1 (Fig. 5d) cells.

**Fig. 6:**
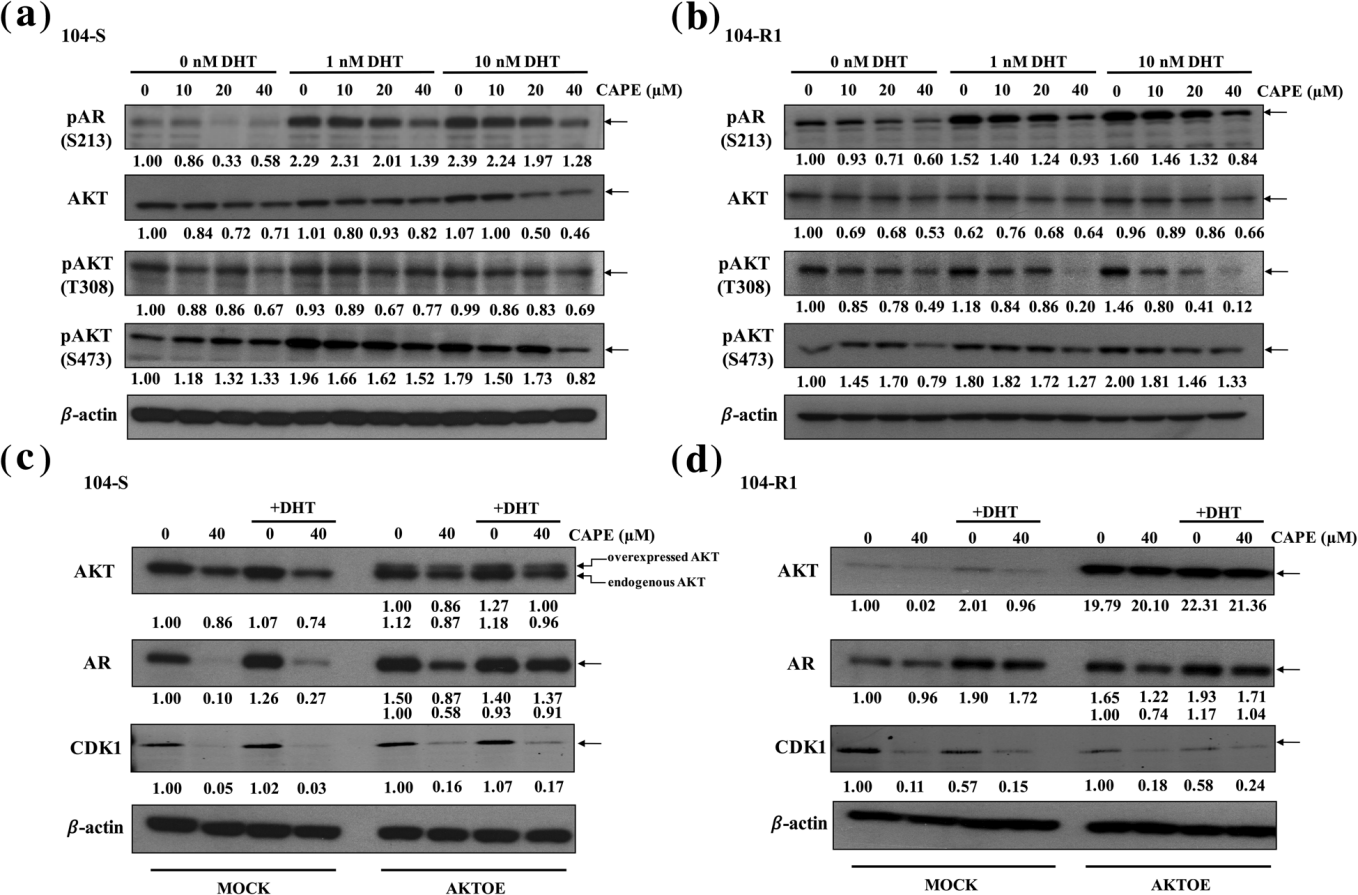
Phosphorylation of AR Ser 213 and AKT signaling pathway were suppressed by CAPE treatment. Cells treated with CAPE and DHT for 48 h were harvested for Western blotting analysis. The expression level of phospho-AR Ser213, AKT, phospho-AKT T308 and phospho-AKT S473 were determined in LNCaP 104-S cells (**a**) and LNCaP 104-R1 cells (**b**). LNCaP 104-S (**c**) and LNCaP 104-R1 (**d**) cells overexpressing AKT were treated with CAPE and DHT for 48 h and collected lysates to analyze protein expression of AR and CDK1. β-actin was used as loading control

AR protein stability is also regulated by phosphorylation on Ser213, which is regulated by PI3K-AKT signaling. Treatment with DHT induced AR phosphorylation on Ser213 in both LNCaP 104-S (Fig. 6a) and LNCaP 104-R1 (Fig. 6b) cells. CAPE treatment dose-dependently reduced the phosphorylation of Serine 213 on AR (Fig. 6). DHT treatment increased the phosphorylation of AKT on Ser473 and Thr308, but had no effect on total AKT abundance in both LNCaP 104-S (Fig. 6a) and LNCaP 104-R1 (Fig. 6b) cells. However, CAPE treatment reduced protein expression level of total AKT, phospho-AKT Ser473, and phospho-AKT Thr308 in both LNCaP 104-S and LNCaP 104-R1 cells (Fig. 6a, b). We examined if overexpression of AKT can rescue the reduction of AR protein under CAPE treatment. Interesting, overexpression of AKT not only increased AR protein level, but also hindered the suppressive effect of CAPE on AR protein abundance in LNCaP 104-S (Fig. 6c) and LNCaP 104-R1 cells (Fig. 6d). Overexpression of AKT did not affect protein level of CDK1. Inhibition of CDK1 protein level was more dramatic than inhibition of AKT protein level by CAPE treatment (Fig. 6c, d). Androgen suppressed CDK1 protein expression in LNCaP 104-R1 cells but not in LNCaP 104-S cells, this is because that the proliferation of LNCaP 104-R1 cells is not dependent on androgen but is suppressed by androgen [27].

We performed animal study to determine if CAPE treatment reduces protein expression level of AR and CDK1 in vivo. Compared to tumors in control nude mice, CAPE treatment (15 mg/kg CAPE via intraperitoneal injection, twice per weeks) significantly reduced AR protein expression level (Additional file 1: Figure S1A, B). CAPE slightly decreased protein abundance of CDK and total AKT, although the difference is not statistically significant.

## Discussion

In this study, we observed that CAPE dose-dependently suppressed the transcriptional activity of AR and protein expression of AR target gene PSA. We discovered that CAPE reduced protein level but not mRNA level of AR in PCa cells. CAPE suppressed the phosphorylation and activity of AKT, thus reduced the phosphorylation of Serine 213 on AR. CAPE also inhibited the phosphorylation of CDK1 kinase, which in turn decreased the phosphorylation of Ser81 on AR. The decrease of Ser213 and Ser81 on AR reduced the stability of AR, and therefore lessened the protein level of AR. Additionally, CAPE treatment reduced the phosphorylation of Ser308 on AR, which then suppressed AR transcriptional activity.

AR phosphorylation plays a critical role in regulating AR function and AR stability. Kinase Src phosphorylates Tyr534 on AR, which regulates AR transcription, PCa cell proliferation, and development of CRPC [28]. Phosphorylation of Ser650 on AR is regulated by stress kinase signaling, and Ser650 antagonizes AR transcription and regulates AR export [29]. Androgen treatment elevates phosphorylation of Serine 16, 81, 256, 308, 424, and 650 on AR of LNCaP cells [30]. Phosphorylation of the AR on Ser308 by CDK1 during mitosis regulates localization and transcriptional activity of AR [25]. AKT phosphorylates Ser213 on AR [31], which promotes AR signaling and CRPC phenotype [32]. Phosphorylation at Ser81 on AR has been reported to stabilize AR and increase the protein expression of AR, the phosphorylation on site is regulated by CDK1 [33] and CDK5 [34]. For certain CRPC cells, elevation of CDK1 activity is a mechanism to increase AR expression and stability in response to low androgen levels in androgen-deprivation therapy [33]. Mutation of S81A on AR blocks its interaction with CDK5, reduces nuclear localization of AR, destabilizes protein level of AR, and decreases proliferation of PCa cells [34]. We observed that CAPE suppressed the expression and activity of AKT and CDK1, which in turn reduced the phosphorylation of Ser213 and Ser81 on AR, respectively. The reduction of Ser 213 and Ser 81 on AR then decreased AR transcription, AR signaling, and AR stability.

It is unclear that if CAPE interacts directly with AR or not. We used computer simulation to investigate the possibility of interaction between CAPE and AR. We first estimate the ability of the molecules to cross the cell membrane. According to the computer simulation, CAPE can cross the cell membrane similar to DHT and antiandrogen bicalutamide (Additional file 2: Figure S2). As we only have information of AR ligand binding domain (LBD), we determined if CAPE binds AR LBD using sophisticated Bayesian statistics to calculate the nuclear receptor ligand score of DHT, CAPE and bicalutamide. Our results revealed that binding between AR LBD and CAPE is much weaker as compared to the binding between AR LBD with DHT or bicalutamide (Additional file 3: Figure S3). Next, we performed fluorescence polarization (FP) AR competition assay to determine the binding affinity between CAPE and AR LBD. The IC_50_ for DHT, bicalutamide, and CAPE to bind AR is 22.3 nM, 183.7 nM, and 1.32 × 10^5^ nM, respectively, indicating that the binding between CAPE and AR is very weak (Additional file 4: Figure S4, Material and Methods for supplemental figures are listed in Additional file 5). The dose of CAPE we used in this study was 10–40 μM. Within this dose range, according to the FP result, CAPE can bind AR but the interaction is probably neglectable. We therefore believe that CAPE regulates phosphorylation of AR mainly through regulation of AKT and CDK1.

## Conclusions

In conclusion, CAPE treatment reduced AR stability and suppressed transcriptional activity of AR in PCa cells, implying the possibility of using CAPE as a treatment for advanced PCa.

## Additional files

Additional file 1: **Figure S1**. CAPE treatment suppressed protein level of AR, CDK1 and AKT of LNCaP 104-R1 xenografts in nude mice. (A) Tumor tissue was lysed and determined by western-blotting. (B) Protein level of AR, CDK1 and AKT was quantitated by ImageJ software. (TIFF 4670 kb)

Additional file 2: **Figure S2**. The hydrophobicity of three compounds (DHT, CAPE and bicalutamide) was analyzed by Druglikeness software. The logP value revealed drug diffusion permeability. Molinspiration druglikeness software is an on-line service software which can be used to compare various molecule properties and structure features which determine whether a particular molecule is similar to the known drugs (https://www.molinspiration.com/docu/miscreen/druglikeness.html). We used this software to compare the hydrophobicity of DHT, CAPE and bicalutamide. (TIF 3862 kb)

Additional file 3: **Figure S3**. The nuclear receptor ligand score of three compounds (DHT, CAPE and bicalutamide) was analyzed by sophisticated Bayesian statistics. Molinspiration druglikeness software was used (with sophisticated Bayesian statistics) to compare the ability of DHT, CAPE, and bicalutamide to bind AR. This software compare the structures of representative ligands active on the particular target with structures of inactive molecules and to identify substructure features typical for active molecules (https://www.molinspiration.com/docu/miscreen/druglikeness.html). The values revealed the ability to bind with AR. (TIF 5090 kb)

Additional file 4: **Figure S4**. The binding ability of AR-ligand binding domain with DHT, CAPE and Bicalutamide was determined by AR competitor assay. AR competitor assay was performed with the PolarScreen AR Competitor Assay kit (Thermo Fisher Scientific) following the manufacturer’s protocol. Reaction plate was incubated for 6 h. Fluorescence polarization was measured by SpectraMax Paradigm Reader and the data was analyzed by Graphpad software. The fluorescence polarization was measured to predict the IC_50. (TIFF 1466 kb)_

Additional file 5: Supplemental Material and Methods. (DOCX 16 kb)

## Data Availability

Due to our internal policy, raw data cannot be shared.

## Abbreviations

AR: Androgen receptor
ARE: Androgen-response elements
CAPE: Caffeic acid phenethyl ester
CHX: Cycloheximide
CRPC: Castration-resistant prostate cancer
CS-FBS: Charcoal-stripped fetal bovine serum
DHT: Dihydrotestosterone
FP: Fluorescence polarization
HSPs: Heat-shock proteins
LBD: Ligand binding domain
PCa: Prostate cancer
PSA: Prostate specific antigen
