# Supplementary figures and images for: Caffeic acid phenethyl ester suppresses androgen receptor signaling and stability via inhibition of phosphorylation on Ser81 and Ser213
Source: Cell Commun Signal. 2019 Aug 20;17:100. doi: 10.1186/s12964-019-0404-9 (PMC6700801; doi:10.1186/s12964-019-0404-9)

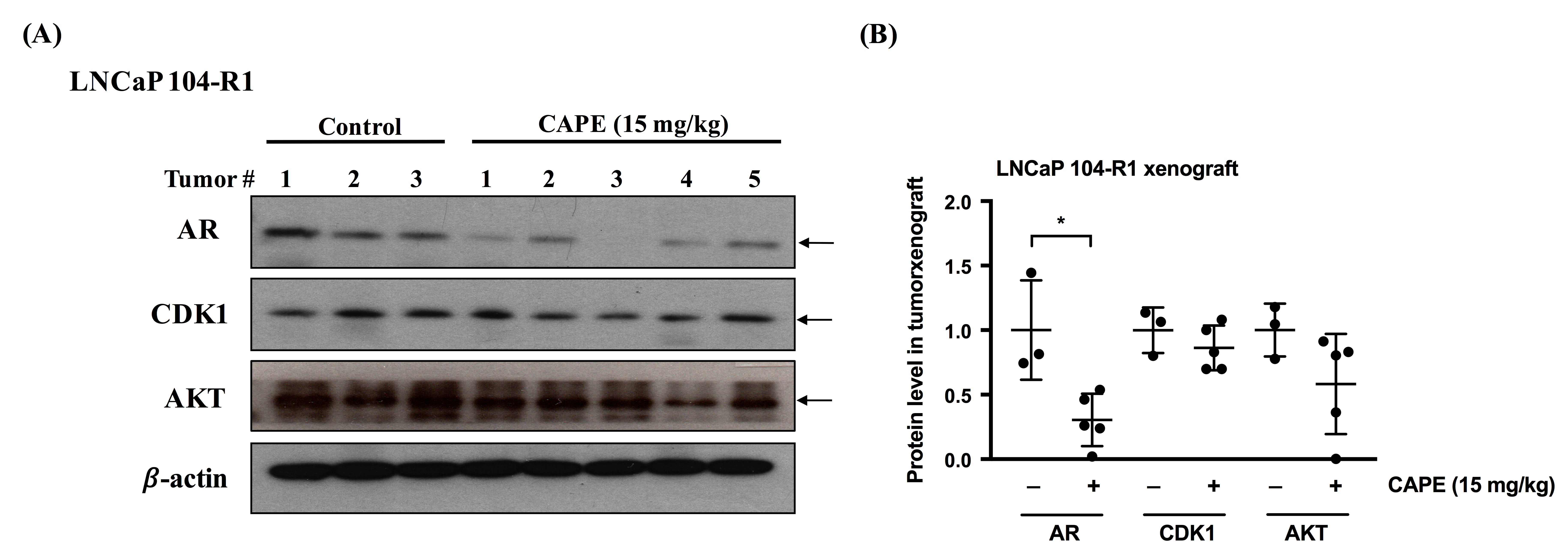

Supplement: Supplementary file 1 — Figure S1. CAPE treatment suppressed protein level of AR, CDK1 and AKT of LNCaP 104-R1 xenografts in nude mice. (A) Tumor tissue was lysed and determined by western-blotting. (B) Protein level of AR, CDK1 and AKT was quantitated by ImageJ software. (TIFF 4670 kb) [file 12964_2019_404_MOESM1_ESM.tiff]

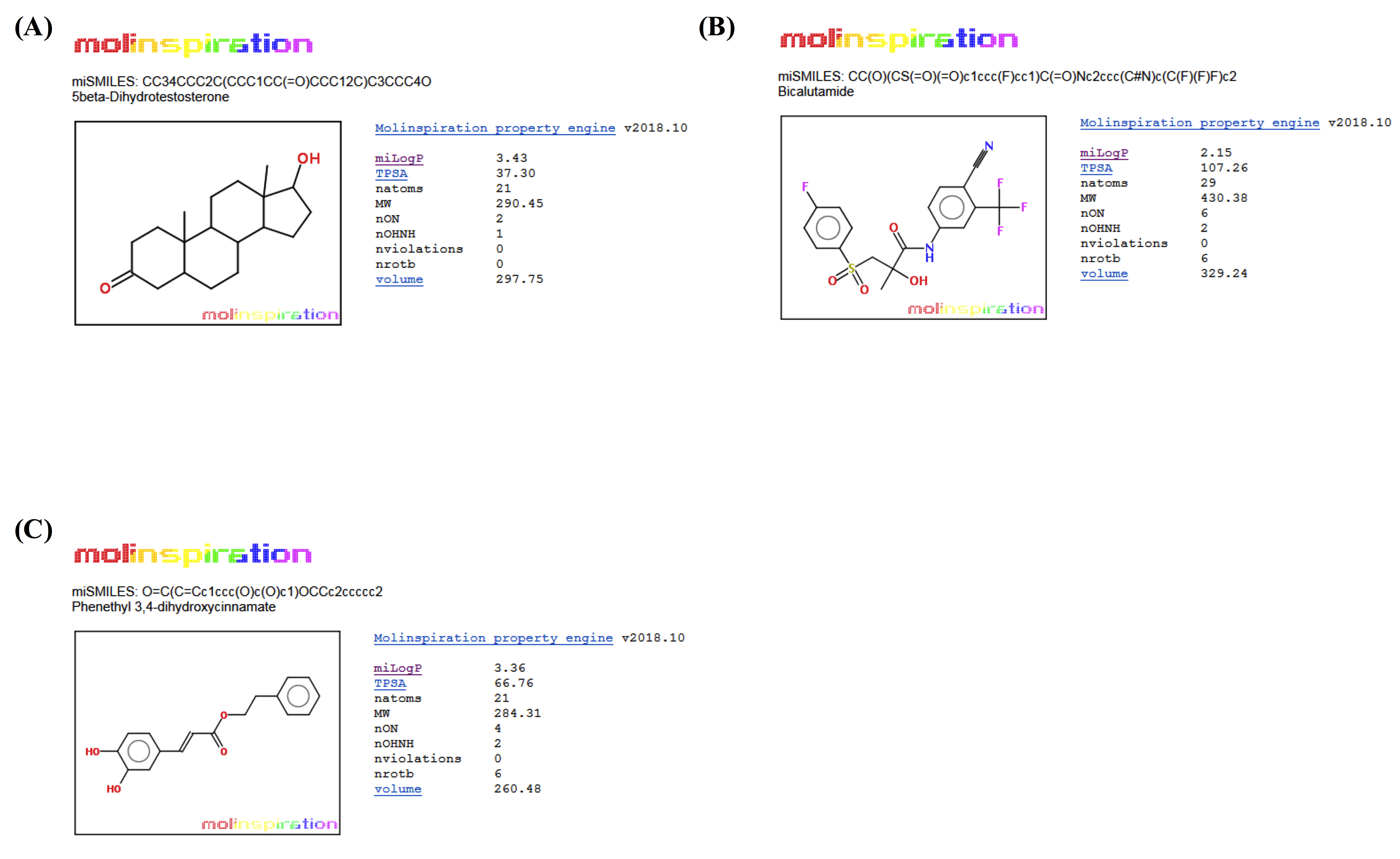

Supplement: Supplementary file 2 — Figure S2. The hydrophobicity of three compounds (DHT, CAPE and bicalutamide) was analyzed by Druglikeness software. The logP value revealed drug diffusion permeability. Molinspiration druglikeness software is an on-line service software which can be used to compare various molecule properties and structure features which determine whether a particular molecule is similar to the known drugs (https://www.molinspiration.com/docu/miscreen/druglikeness.html). We used this software to compare the hydrophobicity of DHT, CAPE and bicalutamide. (TIF 3862 kb) [file 12964_2019_404_MOESM2_ESM.tif]

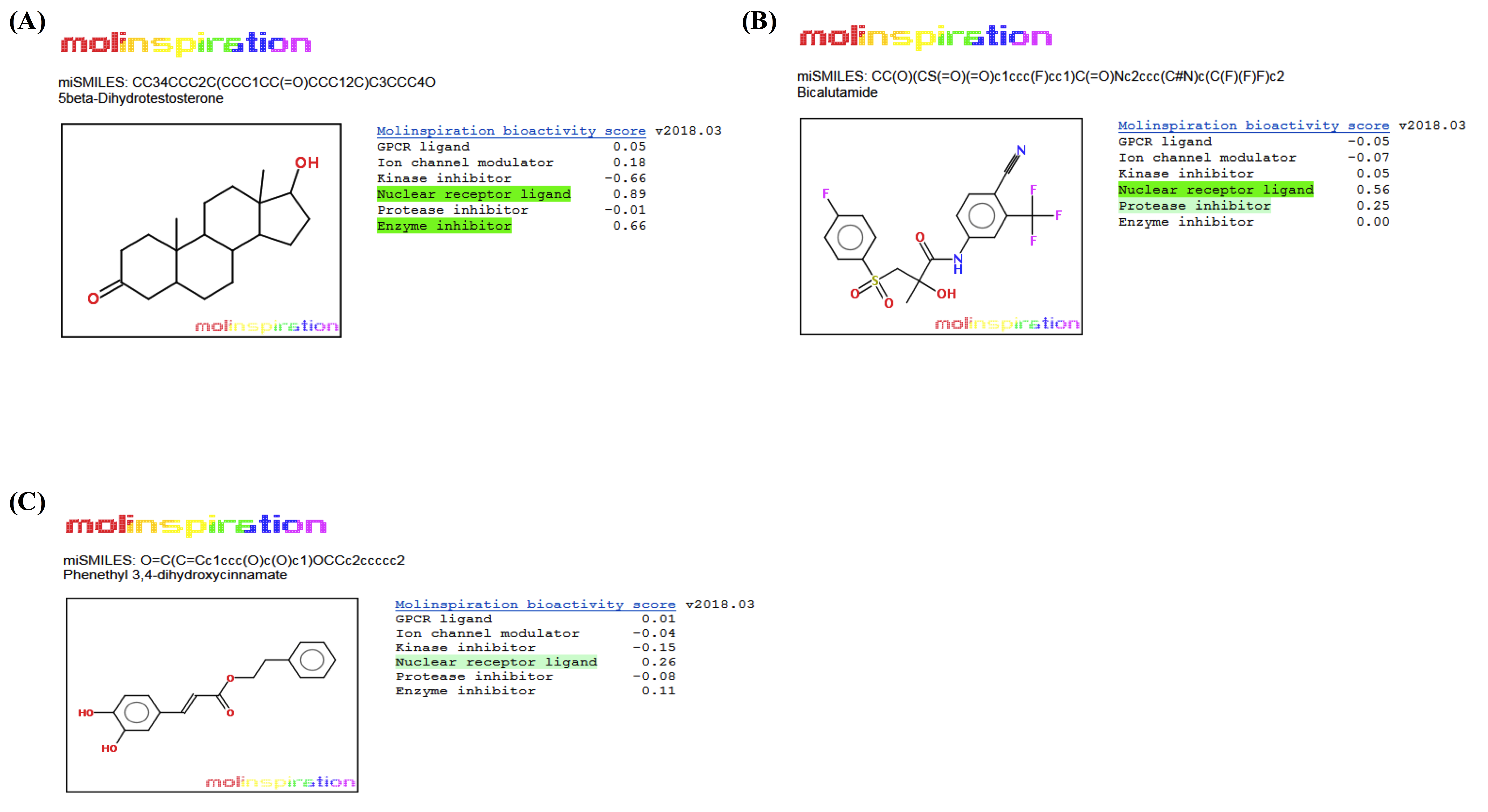

Supplement: Supplementary file 3 — Figure S3. The nuclear receptor ligand score of three compounds (DHT, CAPE and bicalutamide) was analyzed by sophisticated Bayesian statistics. Molinspiration druglikeness software was used (with sophisticated Bayesian statistics) to compare the ability of DHT, CAPE, and bicalutamide to bind AR. This software compare the structures of representative ligands active on the particular target with structures of inactive molecules and to identify substructure features typical for active molecules (https://www.molinspiration.com/docu/miscreen/druglikeness.html). The values revealed the ability to bind with AR. (TIF 5090 kb) [file 12964_2019_404_MOESM3_ESM.tif]

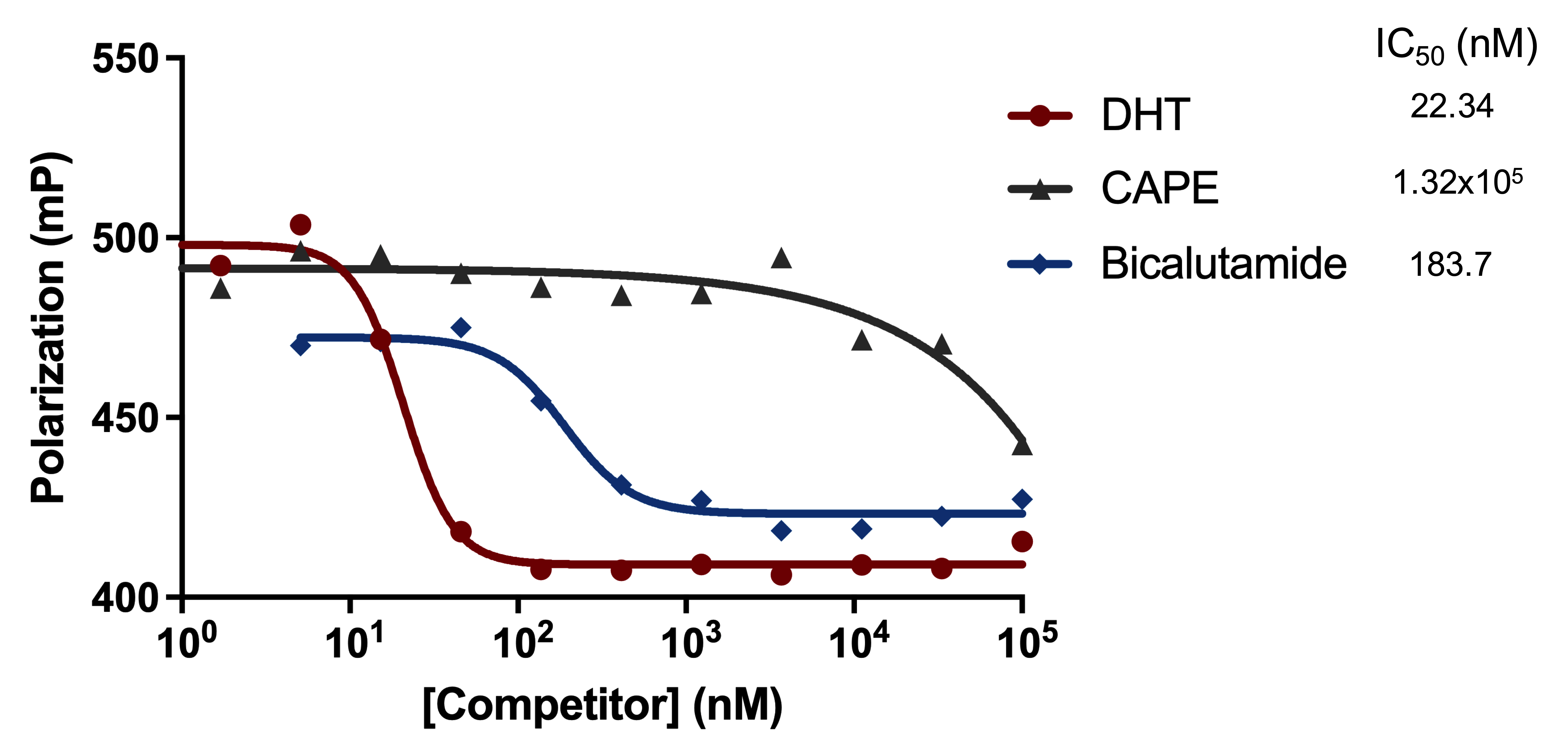

Supplement: Supplementary file 4 — Figure S4. The binding ability of AR-ligand binding domain with DHT, CAPE and Bicalutamide was determined by AR competitor assay. AR competitor assay was performed with the PolarScreen AR Competitor Assay kit (Thermo Fisher Scientific) following the manufacturer’s protocol. Reaction plate was incubated for 6 h. Fluorescence polarization was measured by SpectraMax Paradigm Reader and the data was analyzed by Graphpad software. The fluorescence polarization was measured to predict the IC50. (TIFF 1466 kb) [file 12964_2019_404_MOESM4_ESM.tiff]
