# Supplementary material for: Caffeic acid phenethyl ester suppresses androgen receptor signaling and stability via inhibition of phosphorylation on Ser81 and Ser213
Source: Cell Commun Signal. 2019 Aug 20;17:100. doi: 10.1186/s12964-019-0404-9 (PMC6700801; doi:10.1186/s12964-019-0404-9)
Supplement: Supplementary file 5 — Supplemental Material and Methods. (DOCX 16 kb) [file 12964_2019_404_MOESM5_ESM.docx]

**Supplemental Material and Methods**

**Tumor xenograft.** Experiments involving mice were approved by National Health Research Institutes Institutional Animal Care and Use Committee (NHRI-IACUC-101115-A). The study was carried out in strict accordance with the recommendations in the Guide for the Care and Use of Laboratory Animals of the National Institutes of Health. 5-week-old male mice were castrated and rested for a week. Mice were injected subcutaneously in both flank with 1×10^6^ LNCaP 104-R1 cells and suspended in 100 μL PBS mixed with 100 μL of Matrigel (BD Bioscience). 8 tumors in 6 mice grew up. Mice were randomly separated into control group (3 tumors in 3 mice) and CAPE treatment group (5 tumors in 3 mice). CAPE (15 mg/kg) or vehicle were administered to mice by intraperitoneal injection twice a week for five weeks followed by five days a week after average tumor developed to 100 mm^3^. Tumor were collected at the end point and analyzed by western blotting. Principle Investigator was blinded to the group allocation during the experiment an is the only person to analyze the animal result.

**AR competitor assay.** AR competitor assay was performed with the PolarScreen AR Competitor Assay kit (Thermo Fisher Scientific) following the manufacturer’s protocol. Reaction plate was incubated for 6 hours. Fluorescence polarization was measured by SpectraMax Paradigm Reader and the data was analyzed by Graphpad software.

**Antibody datasheet**. Androgen receptor: purchased from Abcam (ab108341, Cambridge, MA, U.S.A); phospho-AR Ser81: purchased from Millipore (07-1375, Burlington, MA, U.S.A); phospho-AR Ser308: purchased from Santa Cruz (sc-26406, Dallas, TX, U.S.A); phospho-AR Ser213: purchased from GeneTex (GTX50182, Irvine, CA, U.S.A); PSA: purchased from DAKO/Agilent (A0562, Santa Clara, CA, U.S.A); Lamin A/C: purchased from GeneTex (111677, Irvine, CA, U.S.A); CDK1: purchased from Cell Signaling (9116, Danvers, MA, U.S.A); CDK5: purchased from Cell Signaling (2506, Danvers, MA, U.S.A); CDK9: purchased from Cell Signaling (2316, Danvers, MA, U.S.A); Cyclin B1: purchased from Cell Signaling (4135, Danvers, MA, U.S.A); AKT: purchased from Cell Signaling (9272, Danvers, MA, U.S.A); phospho-AKT Ser473: purchased from Cell Signaling (9271, Danvers, MA, U.S.A); phospho-AKT Thr308: purchased from Cell Signaling (2965, Danvers, MA, U.S.A); β-actin: purchased from Novus (NB600-501, Littleton, CO, U.S.A); GAPDH: purchased from Novus (NB300-322, Littleton, CO, U.S.A).
